# “Something old, something new, something borrowed, and the oioxeny is true”: description of *Plectanocotyle jeanloujustinei* n. sp. (Polyopisthocotylea, Plectanocotylidae) from the MNHN Helminthology collection with novel molecular and morphological data for *P*. *gurnardi* (Van Beneden & Hesse, 1863) (*sensu stricto*) from Sweden

**DOI:** 10.1016/j.ijppaw.2024.100914

**Published:** 2024-02-10

**Authors:** Alice Cappelletti, Chahinez Bouguerche

**Affiliations:** a Department of Life and Environmental Sciences, Università degli Studi di Cagliari, Cagliari, Italy; b IFREMER, Channel and North Sea Fisheries Research Unit, 150 Quai Gambetta, BP 699, F-62 321, Boulogne-sur-Mer, France; c ANSES, Laboratory for Food Safety, 62200, Boulogne-sur-Mer, France; d Department of Zoology, Swedish Museum of Natural History, Box 50007, SE-104 05, Stockholm, Sweden

**Keywords:** Polyopisthocotylea, *Plectanocotyle*, Gurnard, Triglidae, Mitochondrial DNA, Morphology, Coast of Sweden, Mediterranean, Museum, Collection

## Abstract

Natural history museums worldwide house billions of apposite specimens, offering the potential for cost-free parasitological datasets. Herein, we provide novel morphological and molecular data (28S and *cox*1) for the polyopisthocotylean *Plectanocotyle gurnardi sensu stricto* from the type-host *Eutrigla gurnardus* from Sweden based on newly collected specimens from the Northeast Atlantic, and specimen from T. Odhner's collections at the Swedish Museum of Natural History (Stockholm, Sweden). The newly generated 28S sequences of *P. gurnardi* from *E. gurnardus* from the Northeast Atlantic were identical to those from the Western Mediterranean, and nested in a single clade, suggesting the presence of a single species. A 28S sequences of *P. gurnardi sensu stricto* from Sweden and those from the U.K. (type locality for *P. caudata*) were identical; we confirm that *P. caudata* and *P. gurnardi* are conspecific and formally synonymize them. A single 28S sequence of *Plectanocotyle* sp. from *Chelidonichthys lastoviza* off France differed from *P. gurnardi* from the Northeast Atlantic by 3–4 % and from *P. gurnardi* from France by 3%. *Plectanocotyle* sp. ex *C. lastoviza* off France is clearly not *P. gurnardi*, suggesting an oioxenic specificity of *P. gurnardi* to *E gurnardus*. Careful re-examination of *Plectanocotyle* cf*. gurnardi* from *C. lastoviza* from the Western Mediterranean from the Helminthology collection of Muséum national d'Histoire naturelle (Paris, France) revealed that it differs from all congeners by morphometry (size of clamps, of terminal lappet and its hamuli and uncinuli, and size of atrial spines). The *cox*1 divergences between *P*. cf*. gurnardi* and *P. major*, *P. lastovizae*, and *P. gurnardi sensu stricto* were 10–11 %, 10–11 % and 8 % respectively, falling within the interspecific variations range. *Plectanocotyle* from the Mediterranean is described as a new species, *P. jeanloujustinei* n. sp. We apprise nomenclature problems in *Plectanocotyle* and consider *P. elliptica* a *species inquirenda*.

## Highlights

• We investigate the oioxeny of *Plectanocotyle gurnardi* using integrative taxonomy.
• *Plectanocotyle* cf. *gurnardi* from the Mediterranean differed by morphometry and DNA.
• *Plectanocotyle* cf. *gurnardi* is described as a new species, *P. jeanloujustinei* n. sp.
• We discuss nomenclature problems in the genus *Plectanocotyle.*
• Based on 28S sequences, we synonymized *P. caudata* with *P. gurnardi.*

## Graphical abstract

**Figure:**
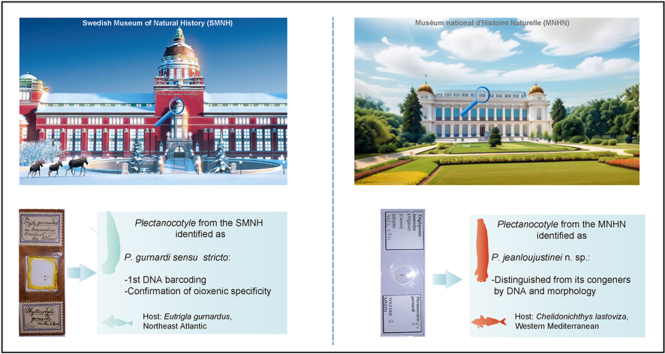

## 1 Introduction

For taxonomists focusing on the systematic study of fish parasites, dissection is commonly the primary technique employed. During dissection, the majority of metazoan parasites, such as Platyhelminthes, associated with vertebrate or invertebrate hosts can be unveiled and sometimes identified using only a light microscope (Harmon et al., 2019). However, certain parasitic flatworms, including the gills parasites, the Polyopisthocotylea Odhner, 1912 are known for being subject to seasonal population dynamics with significant differences in the prevalence rates and peaks in certain seasons (Poulin, 2020). Infestation rates are also often influenced by the study area and host size (Villar-Torres et al., 2018). Hence, the absence of parasites, while advantageous for the host, may temporarily result in missed opportunities to contribute to a better understanding of poorly known parasitic species or even the discovery of novel species. Fortunately, Natural History museums worldwide house billions of relevant specimens accumulated over a century, offering an exceptional potential parasitological dataset for taxonomical studies and various other purposes (Wood and Vanhove, 2023). These collections provide cost-free access, significantly reducing typical expenses related to travel, consumable supplies, and field excursions (Wood et al., 2023).

During an ongoing effort on the study of parasitic Platyhelminthes preserved at the Invertebrate collections at the Swedish Museum of Natural History (SMNH) Stockholm, Sweden (Bouguerche et al., 2023), we found a slide of the polyopisthocotylean *Plectanocotyle gurnardi* (Van Beneden and Hesse, 1863), with some specimens collected by Theodor Odhner 126 years ago (in 1898), from the gills of the type-host the grey gurnard *Eutrigla gurnardus* (Linnaeus, 1758) from Swedish waters of the North Sea, Northeast Atlantic.

This species is of exceptional interest, as it is the only species of the genus for which molecular data are lacking (Ayadi et al., 2022). Most interestingly, Ayadi et al. (2022) reported the presence of *Plectanocotyle* cf. *gurnardi* on a distinct host-streaked gurnard *Chelidonichthys lastoviza* (Bonnaterre, 1788) from a distinct locality, western Mediterranean but no further taxonomic decisions could be made in absence of morphological and molecular data for *P. gurnardi sensu stricto* from its type locality, Northeast Atlantic waters.

Herein, we provide morphological and molecular data for *P. gurnardi sensu stricto* based on museum specimens from the Invertebrate collections at the SMNH and newly collected specimens from the type-host *E. gurnardus* from the Swedish coast of the North Sea, in Northeast Atlantic waters. We also re-examined specimens previously identified as *Plectanocotyle* cf. *gurnardi* preserved at the Helminthology collection at the Muséum national d'Histoire Naturelle (MNHN) Paris, France, and describe a new *Plectanocotyle* species. A molecular and morphometrical differential diagnosis is provided for the newly described species.

## 2 Material and methods

### 2.1 Host and parasite collection

**Fig. 1:**
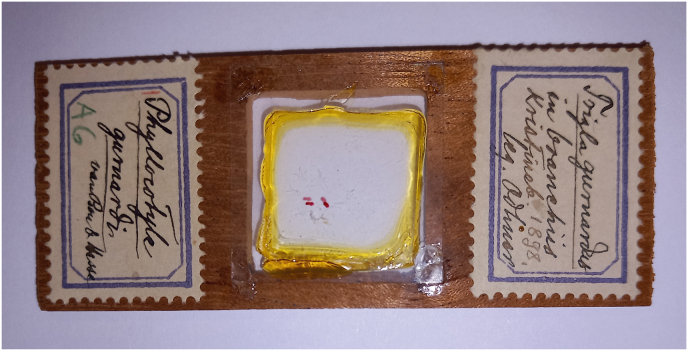
One of the unstudied specimens of *Plectanocotyle gurnardi* (Van Beneden and Hesse, 1863) from the type-host *Eutrigla gurnardus* available for study before this paper, and that hinted the presence of *Plectanocotyle gurnardi* in Swedish waters of the North Sea. The slide containing two specimens of *Plectanocotyle gurnardi* labeled as “*Phyllocotyle gurnardi*”: unstudied specimens collected by Theodor Odhner from the gills of *Eutrigla gurnardus* from Kristineberg, Sweden, Northeast Atlantic.

A slide of *Plectanocotyle gurnardi*, labeled as “*Phyllocotyle gurnardi*” was found in the Invertebrate collections at the Swedish Museum of Natural History (SMNH) in Stockholm, Sweden, and was examined for morphological and morphometrical study. The corresponding labels indicate that the specimens were collected by Theodor Odhner in 1898, from the gills of the type-host the grey gurnard *E. gurnardus* (referred to as *Trigla gurnardus* Linnaeus, 1758) from Kristineberg, Sweden, Northeast Atlantic (Fig. 1). The slide contain two specimens fixed with Malmberg's solution and mounted in Canada balsam.

Parasites for DNA extraction were collected from fish from Swedish waters of the North Sea, Northeast Atlantic (Skagerrak, Kattegat, and Tjärnö). Specimens from Skagerrak and Kattegat were collected during the biannual International Bottom Trawl Survey by the SLU Aqua team as part of the International Bottom Trawl Survey along the Swedish coast within the scope of their research projects and permits and were euthanized and made available for examination. Specimens from Tjärnö were collected in the vicinity of Tjärnö Marine Laboratory, obtained from local fishermen (in dead state) and examined.

Fish were identified using keys (Kullander and Delling, 2012). Polyopisthocotylea were collected from freshly killed fish. The gills were removed, placed in individual petri dishes, and examined.

### 2.2 Morphological methods

**Fig. 2:**
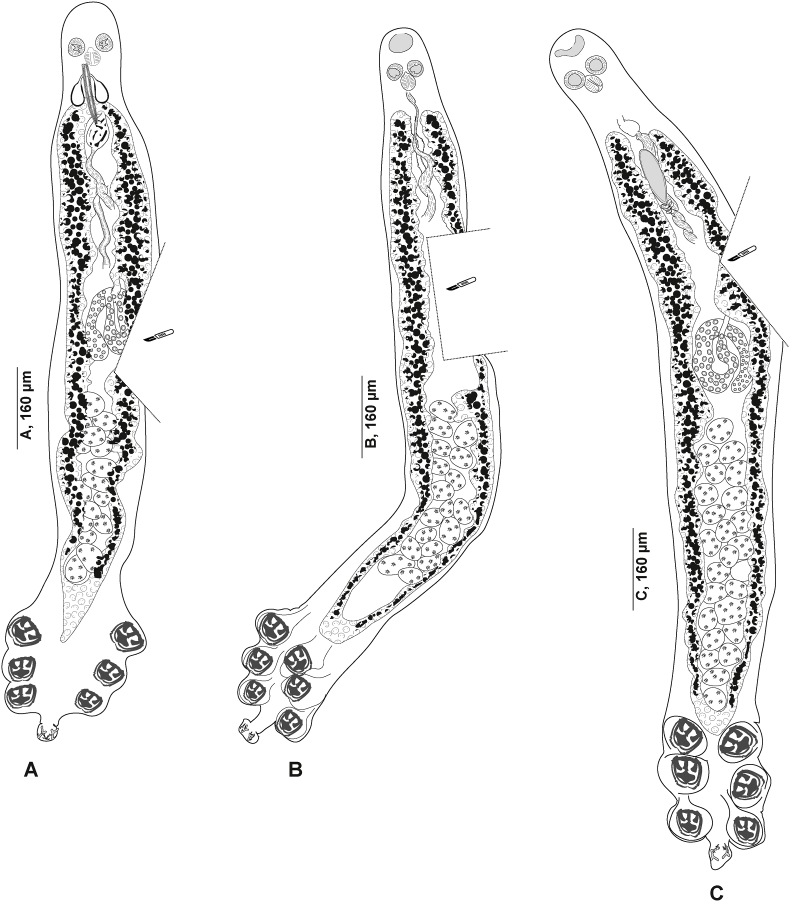
*Plectanocotyle gurnardi* (Van Beneden and Hesse, 1863) *sensu stricto* ex *Eutrigla gurnardus* from the North Sea, Sweden, hologenophores, body lacking only a lateral part; excised used for DNA extraction A, (SMNH 216644). B, (SMNH 216645). C, (SMNH 216646).

**Fig. 3:**
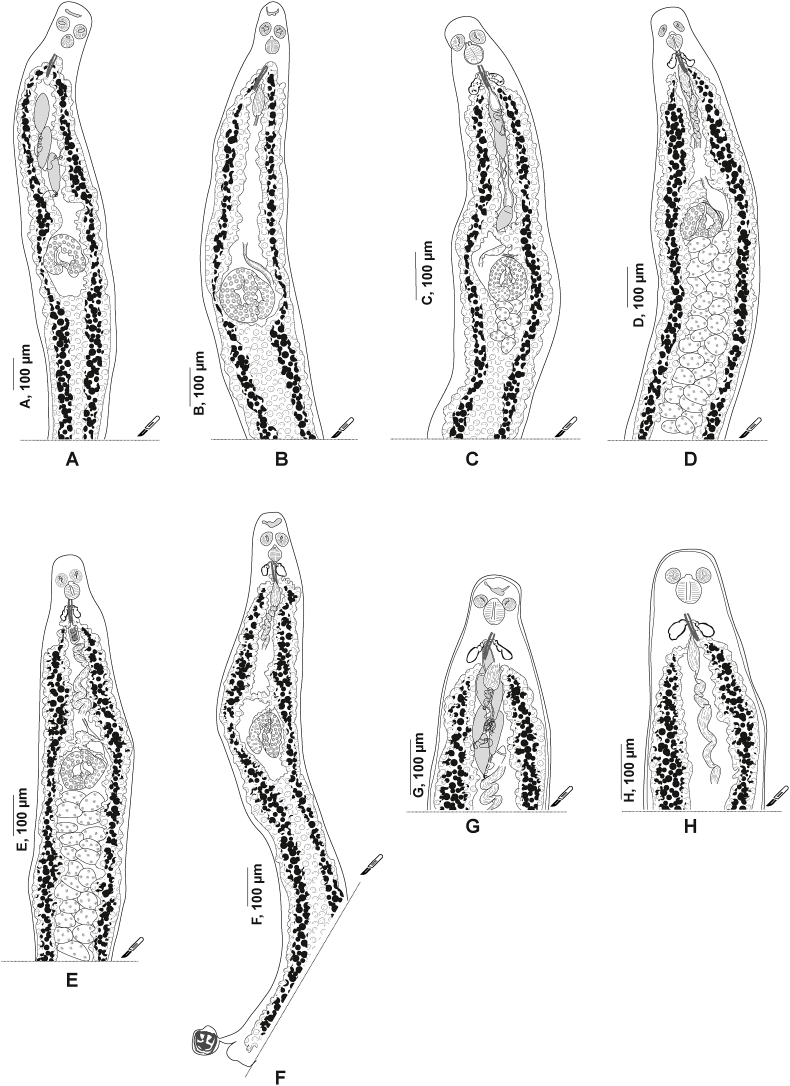
*Plectanocotyle jeanloujustinei* n. sp. ex *Chelidonichthys lastoviza* from the western Mediterranean (Algeria), hologenophores. A-E, Body lacking only the haptor. F. Body lacking a lateral part posterior part. G-H, anterior end of the body including the male copulatory organ. The missing parts were excised and used for DNA extraction. A, (HEL1729). B, (HEL1730). C, (HEL1732). D, (HEL1733). E, (HEL1734). F, (HEL1731). G, (HEL1727). H, (HEL17287).

Newly collected Polyopisthocotylea were heat-killed, fixed without pressure in near-boiling saline, and preserved immediately in 80% ethanol for parallel morphological and molecular characterization. Nine specimens were processed as hologenophores (sensu Pleijel et al. (2008)). Hologenophores of *Plectanocotyle* Diesing, 1850 from Sweden consist of entire specimens, showing taxonomical features (haptor, testes, and male copulatory organ) and lacking only a lateral part (Fig. 2). Hologenophores from the western Mediterranean (off Algeria) consist of specimens lacking only the haptor (Fig. 3A–E); and specimens lacking a lateral posterior part (Fig. 3 F) or anterior end of the body including the male copulatory organ (Fig. 3G and H). Whole-mounts for morphological analysis were stained with acetocarmine or paracarmine, dehydrated in a graded ethanol series, cleared in clove oil, and mounted in Canada balsam. The hologenophores were processed and mounted according to the same methods. Drawings were made through a Nikon Eclipse i80 microscope with DIC (differential interference contrast) and a drawing tube. Drawings were scanned and redrawn on a computer with Adobe Illustrator 2023.

Measurements of whole-mounts and hologenophores are in micrometers and indicated as the range followed by the number of measurements in parentheses. The following abbreviation is used: SMNH, Swedish Museum of Natural History, Stockholm, Sweden; MNHN, Muséum national d'histoire naturelle, Paris, France.

For clamps nomenclature, we followed Llewellyn (1956). For the designation of the ventral and dorsal arms of clamps sclerites, we followed Bouguerche et al. (2021). We followed the terminology as defined by Combes (2003) to describe the host specificity of a parasite with the relatedness of host species: Oioxenic is employed for parasites that exploit a single host species; the parasite is denoted as stenoxenic if it exploits a range of phylogenetically related species and euryxenic if it exploits a range of mutually unrelated species. For high-level terminology of Polyopisthocotylea, we followed the systematics of Brabec et al. (2023) who elevated the former subclasses of "Monogenea" to the level of classes.

### 2.3 Molecular methods

Genomic DNA was extracted from a total of 9 hologenophores, and genetic sequence data were generated for two markers: a partial region of the *cox*1 mitochondrial region (*cox*1 mtDNA) and the large (28S) ribosomal subunit RNA coding region. Total genomic DNA was isolated using the QIAamp DNA Micro Kit (Qiagen). The specific primers JB3 (=COIASmit1) (forward 5′-TTTTTTGGGCATCCTGAGGTTTAT-3′) and JB4.5 (=COI-ASmit2) (reverse 5′-TAAAGAAAGAACATAATGAAAATG-3′) were used to amplify fragments of 411–418 bp of the *cox*1 gene (Bowles et al., 1995; Littlewood et al., 1997). PCR reaction was performed in 20 μl, containing 1 ng of DNA, 1 × CoralLoad PCR buffer, 3 mM MgCl2, 0.25 mM dNTP, 0.15 μM of each primer, and 0.5 units of Taq DNA polymerase (Qiagen). Thermocycles consisted of an initial denaturation step at 94 °C for 2 min, followed by 37 cycles of denaturation at 94 °C for 30 s, annealing at 48 °C for 40 s, and extension at 72 °C for 50 s. The final extension was conducted at 72 °C for 5 min. The sequence was edited with CodonCode Aligner software version 3.7.1, compared to the GenBank database content with BLAST, and deposited in GenBank under accession numbers PP297654, PP297655, PP297658-61, PP297663-65.

A 28S rDNA fragment of 884 bp was amplified using the universals primers C10 (50–ACCCGCTGAATTTAAGCAT–30) and D2 (30–TCCGTGTTTCAAGACGG–50) (Hassouna et al., 1984). PCR reactions were performed in a final volume of 20 mL, containing: 1 ng of DNA, 16CoralLoad PCR buffer, 3 mM MgCl2, 66 mM of each dNTP, 0.15 mM of each primer, and 0.5 units of Taq DNA polymerase (Qiagen). Thermocycles consisted of an initial denaturation step at 94 C for 1 min, followed by 40 cycles of denaturation at 94 C for 30 s, annealing at 60 C, for 30 s, and extension at 72 C for 1 min. The final extension was conducted at 72 C for 7 min (Derouiche et al., 2019). PCR products were visualized on a 1.5% agarose gel, purified, and directly sequenced in both directions on a 3730xl DNA Analyzer 96-capillary sequencers (Applied Biosystems) at Eurofins Genomics. Sequences were edited and assembled using CodonCode Aligner software (CodonCode Corporation, Dedham, MA, USA), and compared to the GenBank database content with BLAST. Sequences from three individual polyopisthocotyleans were obtained and were found to be identical; they were deposited in GenBank under accession numbers PP314021, PP314023, PP326836.

### 2.4 Trees and distances

Phylogenetic analyses were performed using the newly generated sequences of *P. gurnardi* and those of closely related species available in GenBank (Table 1, Table 2). Alignments for each gene region were constructed separately in AliView (Larsson, 2014). The alignment was trimmed to the shortest sequence. Nucleotide substitution models for phylogenetic analyses using the Maximum likelihood method were selected using MEGA11 (Stecher et al., 2020). The Hasegawa-Kishino-Yano model (Hasegawa et al., 1985) with discrete Gamma distribution (HKY + G) was selected for *the cox*1 and The Hasegawa-Kishino-Yano model (Hasegawa et al., 1985) for 28S rDNA. Trees were constructed in MEGA11, with 2000 and 500 replications for *cox*1 and 28S respectively. The Neighbor-joining (NJ) method (Saitou and Nei, 1987) was also used for comparison in MEGA11, with 2000 bootstraps computed for *cox*1, and 28S from the same datasets. P distances and Kimura 2-Parameter (K2P) (Kimura, 1980) were computed from the same datasets with MEGA11.

**Table 1:** Collection data for *cox*1 sequences analyzed in this study. W. M., Western Mediterranean. N.E.A., Northeast Atlantic. * Referred to as *Plectanocotyle* cf. *gurnardi*.

Polyopisthocotylean	Host	Locality	Polyopisthocotylean *cox*1 GenBank	Reference
*Plectanocotyle gurnardi sensu stricto*	*Eutrigla gurnardus*	Skagerrak, Sweden, N.E.A.	PP297654, PP297655, PP297658 (3 sequences)	Present study
*Plectanocotyle gurnardi sensu stricto*	*Eutrigla gurnardus*	Kattegat, Sweden, N.E.A.	PP297659-61, PP297663-65 (6 sequences)	Present study
*Plectanocotyle jeanloujustinei* n. sp.	*Chelidonichthys lastoviza*	Bouharoun, Algeria, W. M.	MW796588-91, MG761763-64	Ayadi et al. (2022) *
*Plectanocotyle lastovizae*	*Chelidonichthys lastoviza*	Bouharoun, Algeria, W. M.	MG761761-62, MW796585	Ayadi et al. (2022)
*Plectanocotyle major*	*Chelidonichthys obscurus*	Bouharoun, Algeria, W. M.	MW796592-94	Ayadi et al. (2022)
*Triglicola obscura*	*Chelidonichthys obscurus*	Bouharoun, Algeria, W. M.	MG761765, MW796584	Ayadi et al. (2022)
*Allogastrocotyle bivaginalis*	*Trachurus picturatus*	Bouharoun, Algeria, W. M.	MN192392	Bouguerche et al. (2019)

**Table 2:** Collection data for 28S sequences analyzed in this study. W.M., Western Mediterranean. N.E.A., Northeast Atlantic. * The polyopisthocotylean parasite annotated on GenBank as “*Plectanocotyloides obscurum*”; the host as “*Aspitrigla obscura*”.

Species	Host	Locality	GenBank ID	Reference
*Plectanocotyle gurnardi sensu stricto*	*Eutrigla gurnardus*	Skagerrak Sweden, N.E.A.	PP314021	Present study
*Plectanocotyle gurnardi sensu stricto*	*Eutrigla gurnardus*	Skagerrak Sweden, N.E.A.	PP314023	Present study
*Plectanocotyle gurnardi sensu stricto*	*Eutrigla gurnardus*	Skagerrak Sweden, N.E.A.	PP326836	Present study
*Plectanocotyle gurnardi*	*Eutrigla gurnardus*	North Sea, U.K., N.E.A.	AF382045	Olson and Littlewood (2002)
*Plectanocotyle gurnardi*	*Eutrigla gurnardus*	Sète, France, W.M.	AF311717	Jovelin and Justine (2001)
*Plectanocotyle* sp.	*Chelidonichthys lastoviza*	Sète, France, W.M.	AF311716	Jovelin and Justine (2001)
*Triglicola obscura* *	*Chelidonichthys obscurus*	Sète, France, W.M.	AF311718	Jovelin and Justine (2001)
*Octomacrum europaeum*	*Alburnoides bipunctatus*	Pasłęka River, Poland	MT441500	Benovics et al. (2021)
*Discocotyle sagittata*	*Salmo trutta*	Isle of Man, U.K., N.E.A.	AF382036	Olson and Littlewood (2002)

**Table 3:** Genetic distances between *cox*1 sequences of Polyopisthocotyleans. Following Ayadi et al. (2022), distances are percentages, and both Kimura-2 and p-distances are indicated. Distances within species are in Italics; intraspecific variations are low, ranging between 0 and 1%. Distances between species (interspecific variations) are higher, ranging between 7 and 8 % to 12–13 %. The most divergent species (in both p-distances and K-2-P) are *P. gurnardi sensu stricto* and *P. major*.

p-distances	*P. gurnardi*	*P. lastovizae*	*P. major*	*P. jeanloujustinei* n. sp.
*Plectanocotyle gurnardi*	*0*			
*Plectanocotyle lastovizae*	11	*0*–*1*		
*Plectanocotyle major*	11–12	7–8	*0*–*1*	
*Plectanocotyle jeanloujustinei* n. sp.	8	10–11	10–11	*0*

Kimura-2 distances	*P. gurnardi*	*P. lastovizae*	*P. major*	*P. jeanloujustinei* n. sp
*Plectanocotyle gurnardi*	*0*			
*Plectanocotyle lastovizae*	11–12	*0*–*1*		
*Plectanocotyle major*	12–13	8–9	*0*–*1*	
*Plectanocotyle jeanloujustinei* n. sp.	8	11–12	10–11	*0*

**Table 4:** Genetic distances between 28S sequences of Polyopisthocotyleans. * Host initially reported as *Trigloporus lastoviza* (GenBank accession number AF311716 (Jovelin and Justine, 2001)). Distances within species are in Italics.

P-distances	*P. gurnardi*ex *Eutrigla gurnardus* off Sweden	*Plectanocotyle* sp. ex *Chelidonichthys lastoviza* off France *
*P. gurnardi* ex *Eutrigla gurnardus* off Sweden	*0*	3–4 %
*P. gurnardi* ex *Eutrigla gurnardus* off U.K.	*0*	3
*P. gurnardi* ex *Eutrigla gurnardus* off France	*0*	3
*Plectanocotyle* sp. ex *Chelidonichthys lastoviza* off France *	3–4 %	

## 3 Results

### 3.1 Molecular characterisation

#### *3.1.1* *Cox*1 sequences

Partial *cox*1 (411–418 nt) sequences were generated for nine isolates of *Plectanocotyle* retrieved from *E. gurnardus* from two localities of the Swedish coasts of the North Sea: off Tjärnö, Skagerrak, and Kattegat. The newly generated *cox*1 sequences were analyzed together with 16 available sequences for plectanocotylids, mainly *Plectanocotyle* and *Triglicola* Mamaev and Parukhin, 1972. The gastrocotylid *Allogastrocotyle bivaginalis* Nasir and Fuentes Zambrano, 1984 (Bouguerche et al., 2019b) was used as an outgroup. The trimmed matrix included 307 positions.

**Fig. 4:**
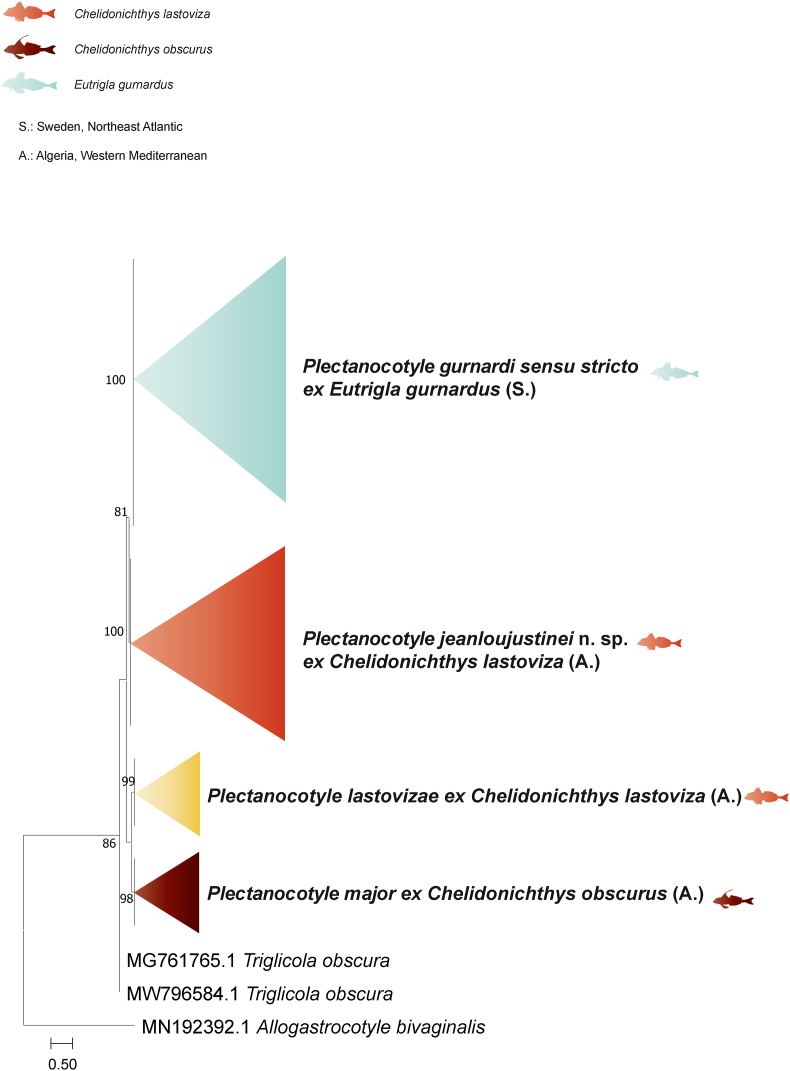
Tree inferred using the ML method based on the *cox*1 sequence data; only bootstrap values higher than 70 are indicated. The newly generated sequences of *Plectanocotyle gurnardi sensu stricto* are indicated in blue. (For interpretation of the references to colour in this figure legend, the reader is referred to the Web version of this article.)

The neighbor-joining and maximum likelihood methods led to similar tree topologies and thus only the ML tree is presented in Fig. 4. The nine generated isolates of *Plectanocotyle* from *E. gurnardus* from Skagerrak and Kattegat clustered together in a well-supported clade, supporting the presence of a single species, *P. gurnardi sensu stricto*.

Similarly, all sequences of *P.* cf. *gurnardi* from *C. lastoviza* from the southern coast of the Western Mediterranean off Algeria nested in a distinct supported sister clade, separated from the *P. gurnardi sensu stricto* clade. *Plectanocotyle* cf. *gurnardi* from the southern coast of the Western Mediterranean off Algeria is thus considered a new distinct species, described herein as *P. jeanloujustinei* n. sp.

Isolates of *P. major* Boudaya, Neifar and Euzet, 2006 from *C. obscurus* (Walbaum, 1792) and of *P. lastovizae* Ayadi, Tazerouti, Gey & Justine, 2022 from *C. lastoviza* from the southern coast of the Western Mediterranean off Algeria nested in separate clades, with high supports for both.

The newly generated sequences of *P. gurnardi sensu stricto* from *E. gurnardus* from the Swedish coasts of the North Sea were identical between them (0% divergence). The newly generated sequences for isolates of *P. gurnardi sensu stricto* from the northeast Atlantic off Sweden differed by 7–9% from *P. jeanloujustinei* n. sp. (*P*. cf. *gurnardi* of Ayadi et al. (2022)) from *C. lastoviza* off Algeria.

The sequences of *P. gurnardi sensu stricto* differed by 11% from *P. major* from *C. obscurus* off Algeria.

The divergence between a sequence of *Plectanocotyle* sp. from *C. lastoviza* off France (AY009169) and those of *P. lastovizae* from the same host off Algeria was 1% suggesting their conspecificty. The divergence between *P. gurnardi sensu stricto* from *E. gurnardus* from Sweden and *P. lastovizae* from *C. lastoviza* off Algeria was 9–10 %.

#### 3.1.2 28S rDNA sequences

Three partial 28S rDNA (921, 927, and 937 nt) sequences were generated for three isolates of *P. gurnardi* from *E. gurnardus* from two localities of the Swedish coasts of the North Sea. The newly generated sequences were compared to other sequences of *Plectanocotyle* available in GenBank. *Octomacrum europaeum* Roman and Bychowsky, 1956 and *Discocotyle sagittat*a Leuckart, 1842 (Diesing, 1850) were used as an outgroup. The trimmed matrix included 447 positions.

**Fig. 5:**
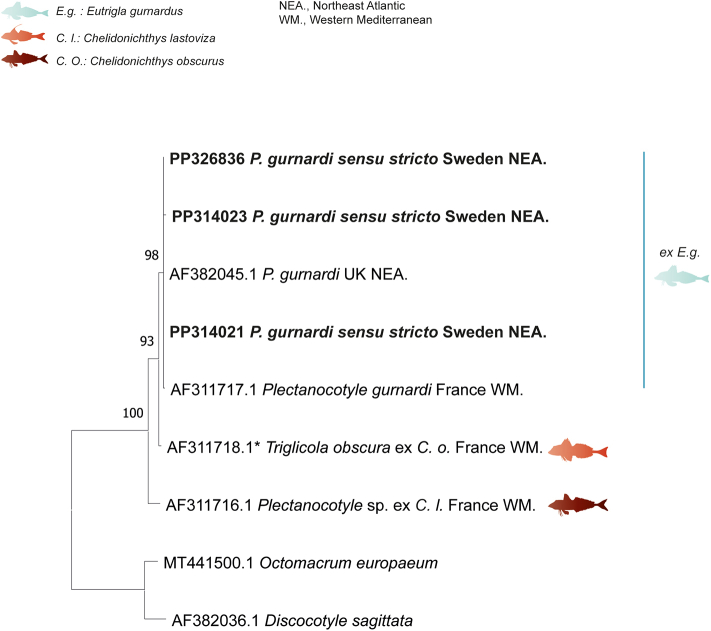
Tree inferred using the ML method based on the 28S rDNA sequence data; only bootstrap values higher than 70 are indicated. The newly generated sequences are indicated in bold. All sequences of *Plectanocotyle gurnardi* from the type-host *Eutrigla gurnardus* clustered in a single clade. * The polyopisthocotylean parasite annotated on GenBank as “*Plectanocotyloides obscurum*”; the host as “*Aspitrigla obscura*”.

The neighbor-joining and maximum likelihood methods led to similar tree topologies and thus only the ML tree is presented in Fig. 5.

**Fig. 6:**
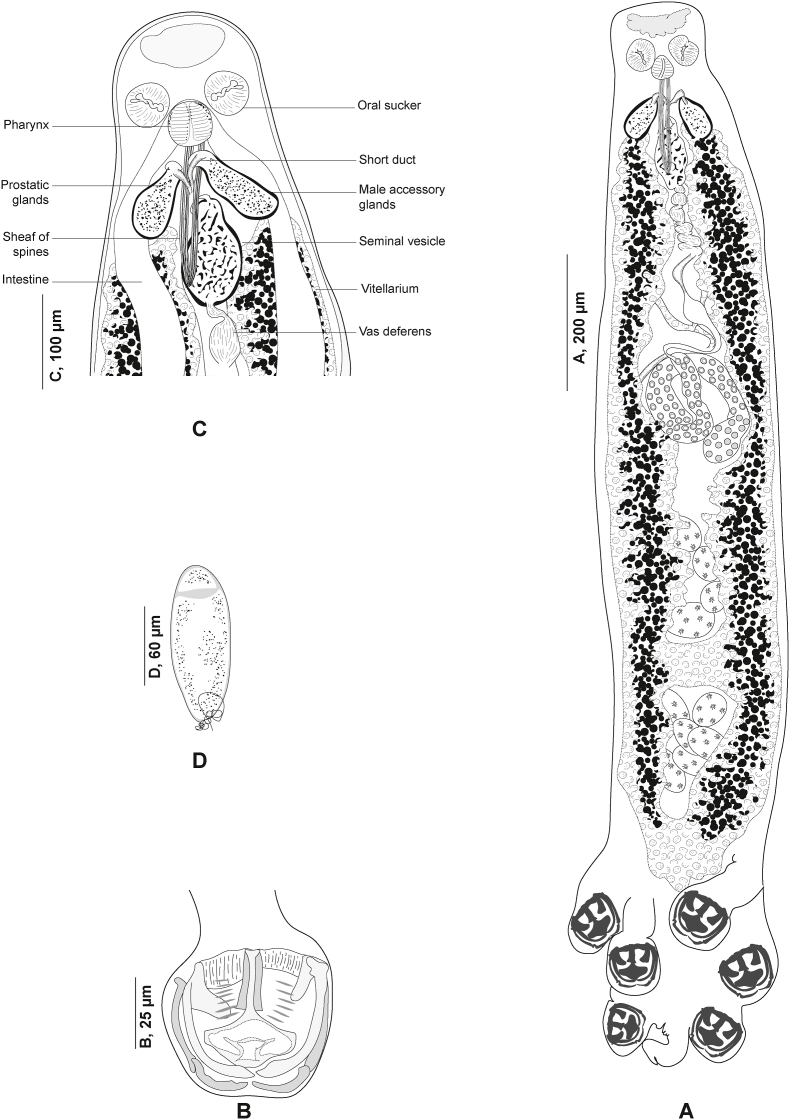
*Plectanocotyle gurnardi* (Van Beneden and Hesse, 1863) *sensu stricto* ex *Eutrigla gurnardus* from the North Sea, Sweden. A, Body, ventral view (SMNH 216639). B, Clamp, ventral view (SMNH 216593). C, Anterior end showing male copulatory organ, ventral view (SMNH 216586). D, Egg, (SMNH 216594).

**Fig. 7:**
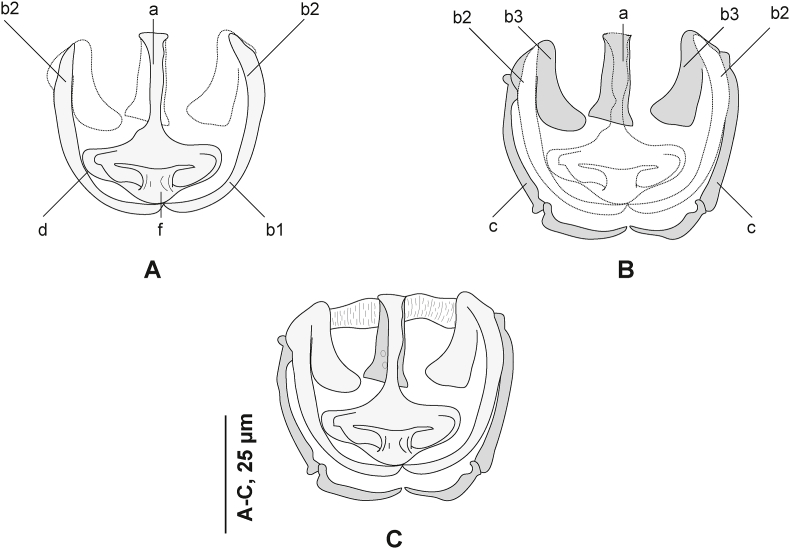
*Plectanocotyle gurnardi* (Van Beneden and Hesse, 1863) *sensu stricto* ex *Eutrigla gurnardus* from the North Sea, Sweden, disposition of clamps sclerites. A, Dorsal jaw. B, Ventral jaw. C, Clamp, ventral view (SMNH 216593).

**Fig. 8:**
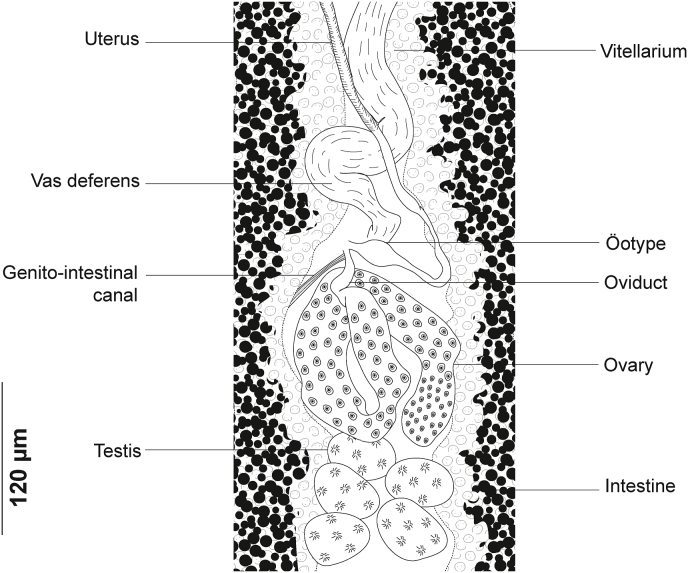
*Plectanocotyle gurnardi* (Van Beneden and Hesse, 1863) *sensu stricto* ex *Eutrigla gurnardus* from the North Sea, Sweden, Detail of the reproductive organs in the region of ovary, ventral view (SMNH 216584).

The newly generated isolates of *P. gurnardi* from *E. gurnardus* from the North Sea from Sweden and the UK, clustered in the same clade with those of *P. gurnardi* from the same host from the western Mediterranean, off France. *Plectanocotyle* sp. from *C. lastovizae* from the northern coast of the Western Mediterranean off France had a basal position.

The newly generated sequences of *P. gurnardi sensu stricto* from *E. gurnardus* from the Swedish coast of the North Sea were identical. The divergence bewteen *P. gurnardi sensu stricto* from Sweden, North Sea and *P. gurnardi* from the same host from the North Sea as well, off the U.K., and those from the northern coast of the Western Mediterranean off France was 0%.

The divergence between *P. gurnardi sensu stricto* from *E. gurnardus* from the North Sea from Sweden and *Plectanocotyle* sp. from *C. lastovizae* from the northern coast of the Western Mediterranean off France was 3–4 %. Similarly, the divergence between *Plectanocotyle* sp. from *C. lastovizae* from the Western Mediterranean off France and *P. gurnardi* from *E. gurnardus* from the same locality was 3 %.

### 3.2 Morphology

**Class Polyopisthocotylea Odhner, 1912**.

**Family Plectanocotylidae Monticelli, 1903**.

**Subfamily Plectanocotylinae Monticelli, 1903**.

**Genus *Plectanocotyle* Diesing, 1850**.

#### 3.2.1 *Plectanocotyle gurnardi* (Van Beneden and Hesse, 1863) *sensu stricto* (Fig. 6, Fig. 7, Fig. 8)

*Synonyms: Plectanocotyle caudata*Lebour, 1908; Llewellyn, 1941); present study.

*Type-host*: *Eutrigla gurnardus* (Perciformes: Triglidae), the grey gurnard (Van Beneden and Hesse, 1863).

*Other hosts*: *Chelidonichthys cuculus* (referred to as *Trigla cuculus*) (Llewellyn, 1941, 1956; Whittington and Kearn, 1989).

*Type-locality*: off Belgium, Northeast Atlantic (Van Beneden and Hesse, 1863).

*Other localities*: Northeast Atlantic: off Plymouth (Llewellyn, 1956; Whittington and Kearn, 1989); off Ireland (Llewellyn, 1941); Skagerrak and Kattegat, Sweden (present study).

*Site on host*: Gills.

*Specimens deposited*:

Specimens with molecular information: Body (lacking only a small lateral part) mounted on a slide, excised lateral part used for molecular analysis: specimens from *Eutrigla gurnardus* off the coast of Sweden, Northeast Atlantic; SMNH 216644, GenBank PP297654, PP314023; SMNH 216645, GenBank PP297655, PP326836; SMNH 216646, GenBank PP297658, PP314021; SMNH 222174, GenBank PP297659; SMNH 222175, GenBank PP297660; SMNH 222176, GenBank PP297661; SMNH 222177, GenBank PP297663; SMNH 222178, GenBank PP297664; SMNH 222179, GenBank PP297665.

Specimens examined for the morphological study, whole mounts: *Plectanocotyle gurnardi* from *Eutrigla gurnardus* from Sweden, Northeast Atlantic, from Tjärnö (SMNH 216644–216646), and from Kattegat (SMNH 216572, SMNH 216575–79, SMNH 216584–98, SMNH 216603, SMNH 216615, SMNH 216623, SMNH 216635).

Material examined for comparison: *P. gurnardi* from *E. gurnardus*, from Kristineberg, Sweden, Northeast Atlantic; from the collection of T. Odhner (SMNH 222180) deposited in the Invertebrates collection in the SMNH.

##### 3.2.1.1 Description

Measurements in Table 5. Measurements of hologenophores provided separately in Table 6. Body elongated, rounded anteriorly (Fig. 6 A); with maximum width at level of ovary. Haptor almost triangular, symmetrical, weakly delimited from body proper, bearing three pairs of clamps. Clamps pedunculated (Fig. 6 B), arranged in two short rows, slightly dissimilar in size anteroposteriorly.

**Table 5:** Measurements of species of *Plectanocotyle*. L., length. W., Width. MCO, male copulatory organ. * Diameter. N.E.A., Northeast Atlantic. W.M., Western Mediterranean.

	*P. gurnardi sensu stricto*	*P. jeanloujustinei* n. sp.	*P. lastovizae*	*P. major*	*P. major*
Host	*Eutrigla gurnardus*	*Chelidonichthys lastoviza*	*Chelidonichthys lastoviza*	*Chelidonichthys obscurus*	*Chelidonichthys obscurus*
Locality	Sweden, N.E.A.	Algeria, W.M.	Algeria, W.M.	Algeria, W.M.	Tunisia, W.M.
Source	Present study	Present study; Ayadi et al. (2022)	Ayadi et al. (2022)	Ayadi et al. (2022)	Boudaya et al. (2006)
Body L.	1681 (1027–2427, n = 25)	2330 (1900–2800, n = 24)	2120 ± 350 (1100–3000, n = 31)	4020 (3380–4400, n = 5)	1841 (1500–2300, n = 11)
Body W.	287 ± 90 (130–475, n = 32)	366 (250–430, n = 24)	310 ± 50 (200–430, n = 31)	472 (370–850, n = 5)	442 (210–600, n = 11)
Oral suckers	51 ± 11 (30–70, n = 33)	41 (29–53, n = 21)	38 (30–46, n = 15)	60 (58–62, n = 4)	48 (40–60, n = 22) *
	× 43 ± 9 (28–64, n = 33)	× 30 (20–73, n = 21)	× 34 (25–45, n = 15)	× 59 (55–62, n = 4)	
Pharynx	45 ± 10 (30–70, n = 32)	60 (50–77, n = 21)	48 (38–60, n = 15)	69 (68–70, n = 4)	45 (30–70, n = 24) *
	× 41 ± 9 (2–61, n = 32)	× 56 (45–70, n = 21)	× 42 (30–56, n = 15)	× 69 (67–70, n = 4)	
Clamp 1	91 (51–115, n = 20)	94 (80–103, n = 10) × 82 (70–90, n = 8)	125 (90–180, n = 11) × 101 (35–135, n = 11	181 (175–195, n = 5) × 139 (125–160, n = 5)	137 (90–160, n = 17) × 110 (85–140, n = 17)
	× 80 (48–108, n = 20)				
Clamp 2	86 (60–109, n = 24)				
	× 77 (52–106, n = 24)				
Clamp 3	82 ± 10 (59–96, n = 31)				
	× 74 ± 10 (52–94, n = 31)				
Sclerites “a” L.	39 (25–51, n = 27)	53 (42–60, n = 10)	60 (51–70, n = 12)	89 (88–90, n = 5)	80 (75–90, n = 21)
Sclerites “b” L.	65 (45–81, n = 27)	76 (60–84, n = 10)	75 (63–85, n = 12)	127 (123–132, n = 5)	157 (140–180, n = 21)
Sclerites “c” L.	58 (41–72, n = 27)	61 (49–76, n = 10)	63 (52–95, n = 12)	83 (80–90, n = 5)	72 (65–90, n = 21)
Sclerites “d” L.	57 (41–72, n = 27)	27 (20–35, n = 8)	20 (12–26, n = 5)	32 (30–35, n = 5)	67 (55–70, n = 21)
Fair-lead (Fl) L.	27 (16–35, n = 27)	30 (26–32, n = 7)	25 (15–32, n = 11)	28 (27–28, n = 2)	33 (25–40, n = 21)
Terminal lappet	145 (73–302, n = 26)	77 (50–115, n = 10)	265 (160–375, n = 19)	61 (56–75, n = 5)	56 (40–80, n = 13)
	× 42 (23–71, n = 26)	× 52 (40–62, n = 10)	× 42 (25–70, n = 19)	× 40 (36–45, n = 5)	× 42 (30–50, n = 13)
Median hamulus L.	43 (32–59, n = 25)	35 (20–51, n = 10)	54 (45–60, n = 12)	74 (27–86, n = 5)	32.5 (25–40, n = 16)
Lateral hamulus L.	57 (41–82, n = 25)	43 (23–53, n = 10)	54 (42–62, n = 12)	77 (37–88, n = 5)	33 (30–35, n = 16)
Postero-lateral uncinulus L.	14 (8–20, n = 25)	11 (6–15, n = 10)	9 (5–13, n = 7)	17 (8–20, n = 5)	12 (10–15, n = 13)
MCO, Peripheral spine L.	193 ± 31 (144–261, n = 33)	107 (90–125, n = 11)	112 (90–162, n = 19)	113 (112–114, n = 5)	107 (90–120, n = 25)
MCO, Median spine L.	122 (91–149, n = 28)	83 (75–97, n = 11)	78 (57–90, n = 18)	75 (73–76, n = 5)	102 (85–110, n = 21)
Number of testes	21 (17–27, n = 29)	23 (14–35, n = 9)	13 (11–15, n = 15)	20 (19–20, n = 5)	21 (19–22, n = 11)
Testis	64 ± 21 (40–135, n = 33)	69 (50–115, n = 8)	63 (45–83, n = 16)	86 (75–100, n = 5)	57 (30–70, n = 30)
	× 50 ± 15 (26–97, n = 33)	× 68 (45–86, n = 8)	× 65 (40–90, n = 16)	× 73 (50–86, n = 5)	× 76 (50–90, n = 30)
Eggs	150 (136–157, n = 7)	147 (143–150, n = 2)	151 (143–161, n = 10)	–	146 (120–185, n = 25)
	× 49 (43–57, n = 7)

**Table 6:** Measurements of hologenophores of *Plectanocotyle gurnardi* (Van Beneden and Hesse, 1863) (*sensu stricto*) from *Eutrigla gurnardus* from the Northeast Atlantic, generated in the present study. L., length. W., Width. MCO, male copulatory organ.

Specimen	SMNH-216644	SMNH-216645	SMNH-216646	SMNH-222174	SMNH-222175
Body L.	2695	2613	2448	2500	2650
Body W.	–	–	323	349	340
Haptor L.	469	429	402	410	415
Haptor W	431	–	–		
Buccal organ	62 × 61	51 × 47	50 × 49	50 × 49	51 × 48
Pharynx	61 × 58	51 × 47	51 × 41	50 × 40	61 × 58
Clamp 1	109 × 196	106 × 103	107 × 98	105 × 94	108 × 195
Clamp 2	103 × 90	102 × 99	93 × 89	90 × 89	102 × 93
Clamp 3	92 × 77	92 × 84	84 × 83	80 × 84	92 × 78
Sclerites “a” L.	48	44	53	50	46
Sclerites “b” L.	60	56	64	64	62
Sclerites “c” L.	65	60	77	76	64
Sclerites “d” L.	65	67	72	0	67
Sclerites “e” L.	21	22	26	26	24
Fair-lead (Fl) L.	29	23	24	24	26
Terminal lappet	156 × 50	151 × 41	140 × 51	145 × 55	156 × 52
Median hamulus L.	39	25	36	38	39
Lateral hamulus L.	45	41	57	58	46
Postero-lateral uncinulus L.	16	15	18	18	16
MCO, Peripheral spine L.	205	197	266	250	255
MCO, Median spine L.	148	127	190	192	162
Number of testes	23	26	25	25	24
Testis	76 × 59	87 × 73	83 × 55	80 × 55	81 × 59
Eggs	–	–	167 × 46	177 × 45	–

Clamps consisting of two opposable jaws, anterior jaw (Fig. 7 A) and posterior jaw (Fig. 7 B). Ventral arm of median spring *a* T-shaped, long, distal part of *a* with large, short branches of equal size, each limb abutting on lateral sclerites. Dorsal arm of median spring *a* visibly shorter than its ventral arm, distally broad, with few apertures arranged in longitudinal parallel rows. Ventral arm of ventral jaw consisting of two lateral sclerites *b2*, dorsal arm *b3* shorter and curved inwards; *b3* not reaching dorsal arm of median spring (Fig. 3B). Dorsal jaw sclerites *c* longer than ventral, formed by two superposed sclerites; *c* reaching midline on distal side. Muscles connecting *a* and *b2* present on proximal side (Fig. 7 C).

Anchor-bearing terminal lappet present (Fig. 6 C), linguiform, consisting of a slender long outgrowth. Terminal lappet bearing three pairs of anchors: two pairs of hamuli and one pair of uncinuli. Lateral hamuli occasionally twice as long as median hamuli. Median hamulus smaller. Uncinuli small, located between hamuli.

Mouth subterminal, elongated transversally. Oral suckers paired, anteriorly placed, subcircular. Pharynx muscular, globular, median. Oesophagus not observed. Intestinal bifurcation anterior to MCO. Caeca with numerous medial and lateral secondary branches, extending into haptor, up to 2/3 total length of haptor. Caeca not confluent posteriorly.

Testes post-ovarian, occupying intercaecal space extending from ovary to haptor; 17–27 in number. Vas deferens medio-dorsal, wide, sinuous, extending anteriorly along mid-longitudinal body axis. Anteriorly, vas deferens expanded into a weekly distinct seminal vesicle in the anterior region.

In its anterior region, vas deferens ending in a narrow duct opening posteriorly in genital atrium. Genital pore near intestinal bifurcation. Cirrus consisting of a sheaf of long spines. Peripheral spines tightly packed in sections. Most inner spines (median) shorter. Median spines delineating a thin canal, receiving the vas deferens.

Male accessory glands paired, with corresponding reservoirs, located at level of cirrus on each side of the body (Fig. 6 C). Male accessory glands with numerous prostatic glands. Secretions of prostatic glands accumulating in small reservoirs. Each reservoir continued rearward by a short duct. Ducts emerging from reservoirs joined medially, forming a prostatic canal. Prostatic canal entering the bundle of spines and uniting with vas deferens.

Ovary complex begins at level of anteriormost testes (Fig. 8). Proximal section irregularly shaped. Ovary continues anteriorly in midline, reflexes again toward anterior extremity, forming large anterior curve before ending in oviduct. Oviduct joined by vitelline reservoir in midline. Genito-intestinal canal (visible only in certain specimens) extending from oviduct ventrally across proximal end of ovary and entering right intestinal caecum. Oötype spindle-shaped, Mehlis’ glands hardly discernible, visible in few specimens. Uterus in midline. Vitellarium co-extensive with intestinal caeca reaching up to haptor region and extending into haptor. Transverse vitelline ducts paired, united anteriorly and posteriorly; posterior junction of transverse vitelline ducts Y-shaped, conspicuous in most specimens, at level of ovary and ventral to it. Egg fusiform (Fig. 6 D), with a single polar filament. Egg filament short, often coiled.

#### 3.2.2 *Plectanocotyle jeanloujustinein. sp.* (Fig. 9, Fig. 10, Fig. 11)

*Synonym*: *Plectanocotyle* cf. *gurnardi* of Ayadi et al. (2022).

*Type-host*: *Chelidonichthys lastoviza* (Perciformes: Triglidae), the streaked gurnard.

*Type-locality*: off Bouharoun (36 37′24.17″N, 2 39′17.38″E), Algerian coast, Western Mediterranean.

*Site on host*: Gills.

*Type-specimens*: Holotype (MNHN HEL1721), 11 paratypes on slides (MNHN HEL1711–1720, MNHN HEL1722) previously deposited in the collections of the Muséum National d’Histoire Naturelle, Paris, and 8 slides (see below) (MNHN HEL1727–HEL1734) including six with sequences (MNHN HEL1729–HEL1734).

Paratype of specimens with molecular information (hologenophores): specimens from *Chelidonichthys lastoviza* off Algeria, Western Mediterranean: specimens lacking only the haptor MNHN HEL1729, GenBank MW796586; MNHN HEL1730, GenBank MW796587; MNHN HEL1732, GenBank MW796589; MNHN HEL1733, GenBank MW796590; MNHN HEL1734, GenBank MW796591. Specimen lacking a lateral posterior part, MNHN HEL1731, GenBank MW796588. Specimens with only the anterior end of the body including the male copulatory organ MNHN HEL1727, MNHN HEL17287. The missing parts were excised and used for DNA extraction.

*ZooBank registration*: To comply with the regulations set out in article 8.5 of the amended 2012 version of the International Code of Zoological Nomenclature (ICZN), details of the new species have been submitted to ZooBank. The Life Science Identifier (LSID) for the new name *Plectanocotyle jeanloujustinei* is urn:lsid:zoobank.org:act:E3D55AC5-D999-4EDE-984C-BE518126D8C9.

*Etymology*: The specific epithet “*jeanloujustinei*” honors French parasitologist Jean-Lou Justine, Professor and curator at the Muséum National d’Histoire Naturelle, Paris, France for his extensive efforts in studying the systematics and biodiversity of polyopisthocotyleans in the western Mediterranean.

##### 3.2.2.1 Description

**Fig. 9:**
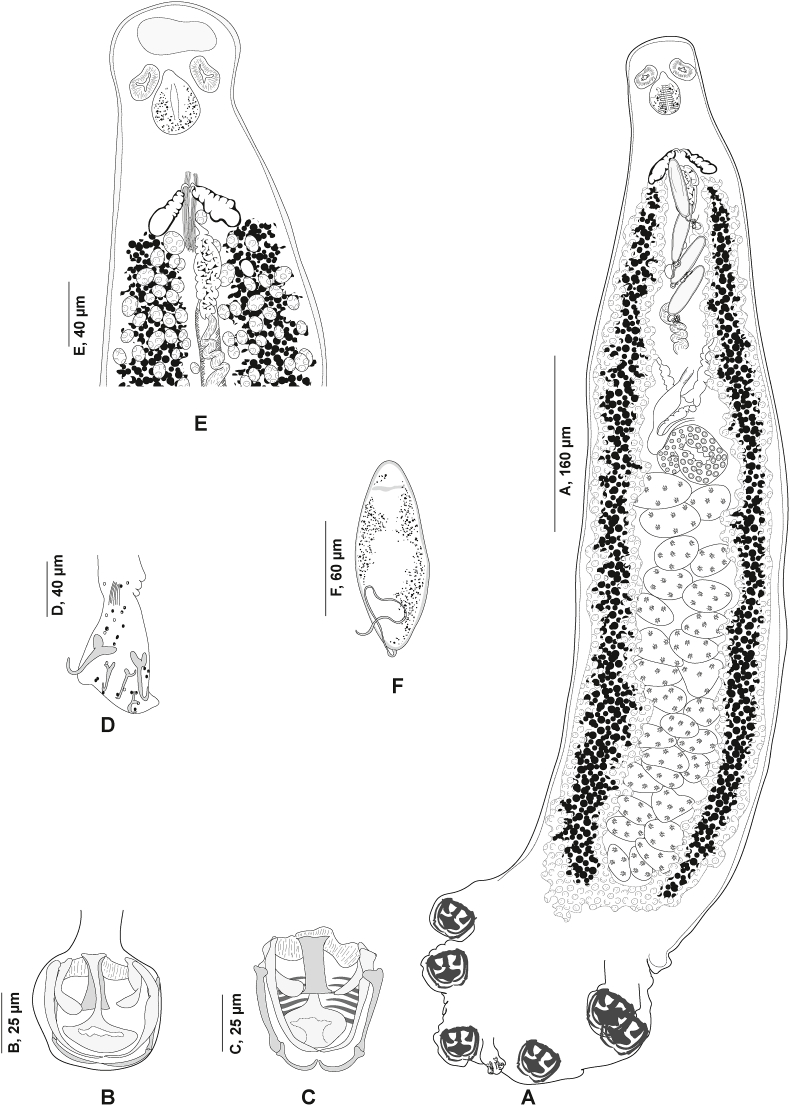
*Plectanocotyle jeanloujustinei* n. sp. ex *Chelidonichthys lastoviza* from the western Mediterranean. A, Body, holotype, ventral view (HEL 1721). B, Clamp, hologenophore, ventral view (HEL 1731). C, Clamp, paratype, dorsal view (HEL 1717). D, terminal lappet, paratype (HEL 1717). E, Anterior end showing male copulatory organ, paratype, ventral view (HEL 1720). F, Egg, paratype (HEL 1714).

**Fig. 10:**
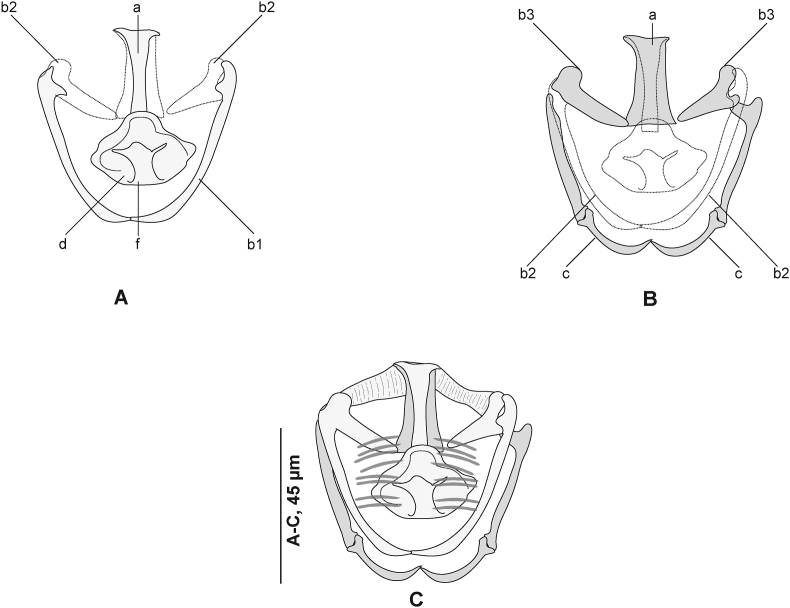
*Plectanocotyle jeanloujustinei* n. sp. ex *Chelidonichthys lastoviza* from the western Mediterranean. Disposition of clamps sclerites. A, Dorsal jaw. B, Ventral jaw. C, Clamp, dorsal view (HEL 1713).

Measurements in Table 5. Body stocky, elongated. (Fig. 9 A). Haptor symmetrical, faintly delineated from body proper, with three pairs of clamps. Clamps pedunculated (Fig. 6 B), arranged in two rows. Clamps consisting of two opposable jaws (Fig. 9 B, C), anterior jaw (Fig. 10 A) and posterior jaw (Fig. 10 B). Organisation of clamps sclerites and muscles similar to that of *P. gurnardi sensu stricto* (Fig. 10 C).

**Fig. 11:**
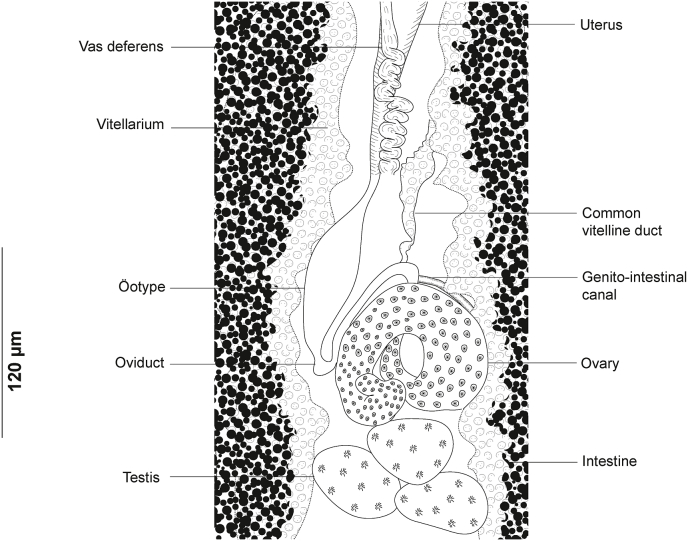
*Plectanocotyle jeanloujustinei* n. sp. ex *Chelidonichthys lastoviza* from the western Mediterranean. Detail of the reproductive organs in the region of ovary, ventral view (HEL 1715).

Anchor-bearing terminal lappet present (Fig. 9 D), linguiform, consisting of a thick short outgrowth. Terminal lappet with three pairs of anchors: two pairs of hamuli and one pair of uncinuli. Lateral hamuli longer than median hamuli. Uncinuli small, located between hamuli.

Mouth subterminal, elongated transversally. Two oval prohaptoral suckers. Pharynx voluminous, pyriform. Intestine with numerous lateral and medial diverticula. Intestinal bifurcation anterior to MCO. Caeca probably not confluent. Testes post-ovarian, intercaecal, mainly in posterior half of body proper, oval, 14–35 in number. Vas deferens sinuous, extending along body midline to male copulatory organ. Vas deferens expanded anteriorly to a globular pyriform seminal vesicle. In its anterior region, vas deferens ending in a narrow thin-walled duct. Cirrus consisting of a sheaf of long slender spines.

Peripheral spines tightly packed. Most inner spines (median) shorter. Median spines delineating a thin canal, receiving the vas deferens.

Male accessory glands paired, with two related smaller reservoirs, located at level of cirrus on each side of the body (Fig. 6 C). Male accessory glands with several prostatic glands. Secretions of prostatic glands collecting in small reservoirs. Each reservoir continued rearward by a short duct. Ducts emerging from reservoirs joined medially, forming a prostatic canal. Prostatic canal entering the bundle of spines and uniting with vas deferens.

Ovary pre-testicular, (Fig. 8). Proximal overlapping most anterior testis. Distal end curved. Oviduct arising from distal end of ovary and connecting common vitelline duct reservoir and oötype. Mehlis’ glands at base of oötype. Uterus originating from oötype and extending anteriorly to genital atrium. Vitellarium follicular, well developed, co-extensive with caeca, extending from level of mal copulatory organ to posterior extremity of body and extending into haptor. Transverse vitelline ducts Y-shaped; dorsal transverse vitelloducts fused approximately at oötype level; common vitelline duct median, fairly long. Eggs fusiform with a single polar filament. Polar filament coiled.

## 4 Discussion

### 4.1 Differential diagnosis of *Plectanocotyle jeanloujustinei* n sp.

*Plectanocotyle jeanloujustinei* n. sp. differs from *P. lastovizae* from the same host *C. lastoviza* by having larger oral suckers (30–70 × 28–64 *vs*. 30–46 × 25–45), larger pharynx (50–77 × 45–70 *vs*. 38–60 × 30–56), and larger clamps (80–103 × 70–90 *vs*. 90–180 × 35–135). *Plectanocotyle jeanloujustinei* n. sp. can also be distinguished from *P. lastovizae* by having a shorter terminal lappet (50–115 *vs*. 160–375), smaller lateral hamuli (23–53 *vs*. 42–62), and smaller median hamuli (20–51 *vs*. 45–60). The two species can be easily differentiated by *P. jeanloujustinei* n. sp. having more testes (14–35 *vs*. 11–15). Additionally, the divergence between the two species in their *cox*1 sequences is 10–11 % in p-distances and 11–12 % in K2P.

*Plectanocotyle jeanloujustinei* n. sp. can be distinguished from *P. major* from *C. obscurus* by having smaller oral suckers (30–70 × 28–64 *vs*. 58–62 × 55–62), smaller clamps (80–103 × 70–90 *vs*. 175–195 × 125–160), larger terminal lappet (50–115 *vs*. 56–75), smaller median hamuli (20–51 *vs*. 27–86), smaller lateral hamuli (23–53 *vs*. 37–88), and smaller postero-lateral uncinuli (6–15 *vs*. 8–20). *Plectanocotyle jeanloujustinei* n. sp. can also be easily distinguished from *P. major* by having more testes (14–35 *vs*. 19–20). Moreover, despite sharing the same locality (Western Mediterranean), the hosts are different (*C. lastoviza* for *P. jeanloujustinei* n. sp. *vs*. *C. obscurus* for *P. major*). Most importantly, *P. jeanloujustinei* n. sp. and *P. major* differ in their *cox*1 sequences by 10–11 % in both K2P and p-distances.

*Plectanocotyle jeanloujustinei* n. sp. differs from *P. gurnardi sensu stricto* from *E. gurnardus* by having smaller oral suckers (29–53 × 20–73 *vs*. 30–70 × 28–64), smaller terminal lappet (50–115 *vs*. 73–302), smaller median hamuli (20–51 *vs*. 32–59), smaller lateral hamuli (23–53 *vs*. 41–82). *Plectanocotyle jeanloujustinei* n. sp. is easily distinguished by having smaller peripheral spines (90–125 *vs*. 144–261) and smaller median spines (75–97 *vs*. 91–149) at the level of the male copulatory organ. *Plectanocotyle jeanloujustinei* n. sp. is easily distinguished from *P. gurnardi sensu stricto* by having more testes (14–35 *vs*. 17–27). Additionally, the type-hosts are different (*C. lastovizae* for *P. jeanloujustinei* n. sp. *vs*. *E. gurnardus* for *P. gurnardi*) and the localities are distinct (Western Mediterranean for *P. jeanloujustinei* n. sp. *vs*. Northeast Atlantic for *P. gurnardi*). Furthermore, the *cox*1 divergence between *P. jeanloujustinei* n. sp. and *P. gurnardi* is 8 % in both K2P and p-distances.

### 4.2 Molecular barcoding for the distinction of *Plectanocotyle* spp.

In the taxonomy of Polyopisthocotylea, it is recurrent to distinguish species by the *cox*1 divergence. Herein, in addition to the morphological differences, the four species of *Plectanocotyle* differ significantly by the divergence in their corresponding *cox*1 sequences **(**Table 3**)** especially given that the recent effort by Ayadi et al. (2022) encompasses molecular data of thoroughly identified specimens (with complete traceability of host i.e., barcoded hosts and deposited hologenophores) of all currently known members of the genus except *P. gurnardi* which was completed in this study.

According to WoRMS (2023), four species have been assigned the genus *Plectanocotyle* (Table 7). Two species are known from Atlantic waters, *P. elliptica* Diesing (1850) (see below) from the white perch *Morone americana* (Gmelin, 1789) (Moronidae) from North America, Northwest Atlantic (Diesing, 1850); and *P. gurnardi* from the grey gurnard *E. gurnardus* (Triglidae) from Belgium, Northeast Atlantic (Van Beneden and Hesse, 1863). Three species occur in Triglidae in the Mediterranean: *P. lorenzii* Monticelli (1899) from *Trigla* sp., off Rovigno, Croatia (Monticelli, 1899) while the remaining two were first described from the Western Mediterranean, with *P. major* Boudaya et al., 2006 from *C. obscurus* from Tunisia (Boudaya et al., 2006) and *P. lastovizae* from *C. lastoviza* off Algeria (Ayadi et al., 2022).

**Table 7:** Host and localities of *Plectanocotyle* spp. from triglid hosts. Note that the record by Scott (1901) (under the name of *Phyllocotyle gurnardi*) in the Nineteenth Annual Report of the Fishery Board for Scotland was afterward recognized by him as belonging to *P. lorenzii* (Scott, 1905). Hosts reported under different names are as follows:^1^ as *Trigla gurnardus*. 2 as *Chelidonichthys gurnardus*.^3^ as *Trigla cuculus*.^4^ as *Aspitrigla cuculus*.^5^ as *Trigla lineata*.

Host and localities	References
**1. *Plectanocotyle gurnardi***	
*Eutrigla gurnardus*	
Belgium, Northeast Atlantic	Van Beneden and Hesse (1863)
Ireland, Northeast Atlantic^1^	Llewellyn (1941)
Plymouth, U.K., Northeast Atlantic	Whittington and Kearn (1989)
Northumberland, U.K., Northeast Atlantic^1^	Lebour (1908)
England, U.K., Northeast Atlantic^1^	Halton and Jennings (1965)
Tunisia, France, Western Mediterranean^2^	Boudaya et al. (2006)
Sweden, Northeast Atlantic	Present study

*Chelidonichthys cuculus*	
Plymouth, Northeast Atlantic^3^	Llewellyn (1956)
Ireland, Northeast Atlantic^3^	Llewellyn (1941)
Plymouth, Northeast Atlantic	Whittington and Kearn (1989)

**2. *Plectanocotyle major***	
*Chelidonichthys obscurus*	
Tunisia, Western Mediterranean	Boudaya et al. (2006): Boudaya et al. (2020)
Algeria, Western Mediterranean	Ayadi et al. (2022)

*Chelidonichthys lastoviza*	
Plymouth, Northeast Atlantic^5^	Llewellyn (1956)

**3. *Plectanocotyle lastovizae***	
*Chelidonichthys lastoviza*	
Algeria, Western Mediterranean	Ayadi et al. (2022)
France, Western Mediterranean (as *Plectanocotyle* sp.)	Jovelin and Justine (2001)

**4. *Plectanocotyle jeanloujustinei* n. sp.**	
*Chelidonichthys lastoviza*	
Algeria, Western Mediterranean	Present study

Generally, the interspecific and intraspecific variations of *cox*1 sequences in Polyopisthocotylea ranged from 0.2 to 5.6% (Bouguerche et al., 2019a, Bouguerche et al., 2019b). The divergence between *P. gurnardi sensu stricto* from the type-host *E. gurnardus* off Sweden (i.e., close to the type locality, northeast Atlantic) and *P. jeanloujustinei* n. sp. (designated as *P.* cf. *gurnardi* by Ayadi et al. (2022) surpassed the interspecific threshold. Moreover, the hosts are different (*C. lastovizae* for *P. jeanloujustinei* n. sp. *vs*. *E. gurnardus* for *P. gurnardi sensu stricto*) and the type localities are distinct (western Mediterranean *vs*. northeast Atlantic).

In the present study, we also generated 28S rDNA sequences for *P. gurnardi sensu stricto* from Sweden. Unfortunately, fewer 28S rDNA sequences of *Plectanocotyle* are available in GenBank and limited to *P. gurnardi* from *E. gurnardus* from the U.K., northeast Atlantic (Olson and Littlewood, 2002) and the same host off France, Western Mediterranean (Jovelin and Justine, 2001); and a sequence designated as *Plectanocotyle* sp. from *C. lastovizae* also from off France, Western Mediterranean (Jovelin and Justine, 2001). All isolates of *P. gurnardi* from the type-host *E. gurnardus* from the North Sea (from Sweden and the U.K.) and the western Mediterranean differed by one and 2 pb respectively, suggesting the presence of a single species *P. gurnardi* on *E. gurnardi*. Thus, the previous record of *P. gurnardi* on its type-host *E. gurnardus* in the western Mediterranean by Jovelin and Justine (2001) is highly likely accurate.

Overall, the presence of Polyopisthocotylea in distinct localities is not unusual and has been previously challenged by *cox*1 barcodes. Consequently, the taxonomic and geographic status of several polyopisthocotyleans has been validated (see (Hossen et al., 2022a, 2022b). Hossen et al. (2022b) demonstrated with *cox*1 barcodes the presence of *Allogastrocotyle bivaginalis* both in Mediterranean waters, off Algeria (report by Bouguerche et al., 2019a, Bouguerche et al., 2019b) and in Australian waters (Victoria) of the southwest Pacific. The authors also demonstrated the presence of *Kuhnia scombri* (Kuhn, 1829) and *Pseudokuhnia minor* (Goto, 1984) in ten locations along the coast of China (recorded by Yan et al. (2016)) and in Australian waters (Victoria) of the southwest pacific (Hossen et al., 2022b). Similarly, the microcotylids *Microcotyle algeriensis* Ayadi, Gey, Justine & Tazerouti, 2016 and *M. merche* Víllora-Montero, Pérez-del-Olmo, Valmaseda-Angulo, Raga & Montero, 2023 has been demonstrated to occur both in the western Mediterranean and northeast Atlantic waters (Víllora-Montero et al., 2023).

Most interestingly, a single 28S rDNA sequence of *Plectanocotyle* sp. from *C. lastoviza* off France differed from *P. gurnardi* from the northeast Atlantic by 3–4 % and from *P. gurnardi* from the same locality, France by 3% despite that the analyzed sequences correspond to a highly conserved region of the nuclear gene (28S rRNA) (Table 4). Hence, *Plectanocotyle* sp. ex *C. lastoviza* off France is clearly not *P. gurnardi*. At that time, neither *P. major* nor *P. lastovizae* were described yet (Ayadi et al., 2022; Boudaya et al., 2006), and the only available species in the genus was *P. gurnardi*, and given the high divergence in 28S rDNA, Jovelin and Justine (2001) were also accurate in not identifying their *Plectanocotyle* sp. as *P. gurnardi*. Unfortunately, there are no *cox*1 sequences of *Plectanocotyle* sp. of Jovelin and Justine (2001) nor 28S rDNA for *P. lastovizae* and so far, *P. gurnardi* is the sole species of the genus for which both *cox*1 and 28S rDNA are available.

Given that *C. lastovizae* from the western Mediterranean hosts two different species, *P. lastovizae* (Ayadi et al., 2022) and *P. jeanloujustinei* n. sp., we cannot ascertain if *Plectanocotyle* sp. of Jovelin and Justine (2001) is *P. lastovizae* or *P. jeanloujustinei* n. sp.

### 4.3 Taxonomy of *Plectanocotyle* spp.

There are several ambiguities surrounding the type-species of the genus, *P. elliptica*, first described from a member of the Moronidae 174 years ago (in 1850), but was not found again since its original description, and its validity was thus questioned (Ayadi et al., 2022; Price, 1961). One might be tempted to argue against this, by referring for example to the diclidophorid *Flexophora ophidii* Prost and Euzet, 1962, first described from the snake blenny *Ophidion barbatum* Linnaeus, 1758 from off Sète, France in 1962 (Prost and Euzet, 1962), never reported since until Bouguerche et al. (2020) redescribed it based on newly collected specimens from the western Mediterranean, 58 years later.

Moreover, Price (1961) suggested that *P. elliptica* was described based on a mutilated specimen, and most importantly, it was suggested that either the host fish had been misidentified or the specimen had been mislabeled as to origin (Price, 1961). Herein, following Ayadi et al. (2022), we consider, *P. elliptica* a *species inquirenda*.

*Plectanocotyle caudata*Lebour, 1908 was described from *E. gurnardus* in the U.K., northeast Atlantic (Lebour, 1908). In the present study, 28S rDNA sequences of *P. gurnardi sensu stricto* from Sweden and those from the U.K., northeast Atlantic (Olson and Littlewood, 2002) were identical between them and nested within a single clade. Thus, following Llewellyn (1941) we consider that *P. caudata* and *P. gurnardi* represent the same species and we formally herein synonymize the two species.

Another gap in the knowledge of the systematics of the genus *Plectanocotyle* was partially related to *P. gurnardi*, first described as *Phyllocotyle gurnardi* Van Beneden and Hesse, 1863. Despite the extensive efforts by Llewellyn (1941) who investigated the taxonomy of *P. gurnardi*, transferred it consequently to the genus *Plectanocotyle,* and described its clamps and its adhesive mechanisms (Llewellyn, 1956), there are no morphometrical data for *P. gurnardi* from the type-host and type locality, and it is the sole species of the genus for which molecular data are lacking. Additionally, morphometric data for *P. gurnardi* from the type-host and type locality were lacking. Consequently, in their revisions of the genus *Plectanocotyle,* Ayadi et al. (2022) took a conservative approach and identified their *Plectanocotyle* from *C. lastovizae* as *P*. cf. *gurnardi*.

Herein, despite the absence of published records of *P. gurnardi* from Swedish waters, the “old” T. Odhner's slide of *P. gurnardi* labeled as “*Phyllocotyle gurnardi*”, with some specimens collected by T. Odhner 126 years ago (in 1898) and found in the Invertebrate collections at the SMNH (Stockholm, Sweden) triggered this investigation as it hinted at the presence of *P. gurnardi* in Swedish waters of the Northeast Atlantic. As indicated in the early efforts by Justine et al. (2013), this study is another example of the importance of Museum collections for modern research. The sampling of the type-host *E. gurnardus* allowed to collect “new” specimens for DNA extraction and to extend morphometrical data. Therefore, we provide a redescription of *P. gurnardi sensu stricto*, and we expanded the molecular database for the genus by providing for the first time *cox*1 and 28S sequences for *P. gurnardi sensu stricto*. Careful examination of the borrowed slides of *P*. cf. *gurnardi* from *C. lastoviza* from the Helminthology collections at the MNHN (Paris, France) revealed that *P*. cf. *gurnardi* from the western Mediterranean differs from *P. gurnardi* from the Northeast Atlantic by morphology and molecules, and *P*. cf. *gurnardi* was thus described as a new species, *P. jeanloujustinei* n. sp. Thus, *Plectanocotyle gurnardi* does not occur on *C. lastovizae* and is oioxenic to *E. gurnardus* while *P. jeanloujustinei* n. sp. is oioxenic to *C. lastovizae*.

Hence, by combining “*Something old*”, “*something new*”, and “*something borrowed*”, we described a new species; and we demonstrated that in *Plectanocotyle*, the “*oioxeny is true*”. To the best of our knowledge, *Plectanocotyle* is the only genus among the Polyopisthocotylea for which all known (or all valid) species have been characterised by both morphology and DNA. *Plectanocotyle jeanloujustinei* n. sp. is the only polyopisthocotylean species that had been differentiated from all its congeners based on *cox*1 sequences as all four *Plectanocotyle* valid species, *Plectanocotyle gurnardi*, *P. major*, *P. lastovizae* and *P. jeanloujustinei* n. sp. were characterised by DNA barcoding.

## Declaration of competing interest

The authors declare that there is no conflict of interest.
